# CT Anatomy of the Lower Respiratory Tracts of the *Testudo graeca*

**DOI:** 10.1002/vms3.71013

**Published:** 2026-07-09

**Authors:** Omid Zehtabvar, Ali Reza Vajhi, Amir Rostami, Hesameddin Akbarein, Somaye Davudypoor, Zahra Sherafat, Seyyed Kamyab Momeni, Seyyed Hossein Modarres Tonekabony

**Affiliations:** ^1^ Anatomy Sector Department of Basic Sciences Faculty of Veterinary Medicine University of Tehran Tehran Iran; ^2^ Department of Surgery and Radiology Faculty of Veterinary Medicine University of Tehran Tehran Iran; ^3^ Department of Internal Medicine Faculty of Veterinary Medicine University of Tehran Tehran Iran; ^4^ Department of Food Hygiene and Quality Control Faculty of Veterinary Medicine University of Tehran Tehran Iran; ^5^ Veterinary Radiologist Graduated from Faculty of Veterinary Medicine University of Tehran Tehran Iran; ^6^ DVM Graduated from Faculty of Veterinary Medicine University of Tehran Tehran Iran; ^7^ DVM Student Faculty of Veterinary Medicine University of Tehran Tehran Iran

**Keywords:** bronchial anatomy, computed tomography, pulmonary volume change

## Abstract

**Background:**

The respiratory system of *Testudo graeca* (spur‐thighed tortoise) has not been fully explored, especially when it comes to the detailed structure of the lungs and bronchi. Understanding these anatomical features, along with how they change when the tortoise repositions its body, is important for both studying reptile biology and for clinical practices.

**Methods:**

In this study, we used high‐resolution computed tomography (CT) to examine the trachea, bronchi and lungs of *T. graeca*. The tortoises were scanned in two different body positions: When their head, neck and limbs were retracted inside the shell (flexed), and when these body parts were extended outside the shell. We measured key anatomical features such as lung volume, lung dimensions (length, width and height), bronchial structure and parenchymal thickness.

**Results:**

CT imaging provided detailed views of the lung and bronchial structures. We found that the lungs of *T. graeca* are divided into distinct lateral and medial sections, with the bronchi branching into secondary bronchi that supply these areas. When the head, neck and limbs were retracted inside the shell, lung volume decreased, primarily in the cranial portion of the lungs. However, the overall lung shape, the branching of the bronchi and the thickness of the lung tissue remained unchanged between the extended and retracted positions. The bifurcation of the trachea and the orientation of the bronchi were consistent in both postures.

**Conclusions:**

This study provides a comprehensive description of the lung and bronchial structures in *T. graeca*. It also shows that while retracting the head, neck and limbs affects lung volume, it does not significantly alter lung morphology or bronchial structure. CT imaging proved to be an effective, non‐invasive method for studying the respiratory system in turtles, especially considering the challenges of accessing their internal structures. These findings offer important information that can guide future studies on respiratory adaptations in reptiles and support their clinical management.

## Graphical abstract

In this study, we used high‐resolution computed tomography (CT) to examine the trachea, bronchi and lungs of *Testudo graeca*. The tortoises were scanned in two different body positions: When their head, neck and limbs were retracted inside the shell (flexed), and when these body parts were extended outside the shell.

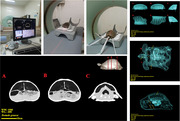

## 1 Introduction

The respiratory system of *Testudo graeca* (the spur‐thighed or Greek tortoise) exhibits distinct anatomical and functional features consistent with those of other reptiles. In contrast to the broncho‐alveolar lung structure typical of mammals, reptilian lungs, including those of *T. graeca*, possess a multicameral, faveolar morphology. This structural design, composed of numerous interconnected chambers or sacs, serves to augment the surface area available for gas exchange, thereby supporting the species‐specific respiratory requirements. Notably, body posture can significantly influence breathing mechanics by altering lung shape and volume, making the assessment of such postural effects a critical component in the clinical evaluation of tortoises presenting with respiratory disease. A recent study showed that static and dynamic lung compliance in terrestrial tortoises is closely related to shell structure and body posture (Oliveira et al. 2024).

Recent advances in non‐invasive imaging, particularly computed tomography (CT), have revolutionized the study of reptilian respiratory systems. This technology enables detailed, three‐dimensional visualization of pulmonary anatomy, allowing for precise quantification of lung volume, internal chamber architecture and overall structural organization without necessitating invasive dissection. For instance, da Silva et al. employed CT to investigate the lung morphology of red‐footed tortoises (*Chelonoidis carbonaria*), establishing comprehensive reference metrics for their pulmonary volume and chamber configuration (Andrés Polanco et al. 2020). Complementing this, Polanco et al. (2020) utilized CT to elucidate the bronchial structure of the same species, significantly enhancing the understanding of pulmonary branching patterns (Polanco et al. 2020). Furthermore, a recent study on loggerhead sea turtles (*Caretta caretta*) has demonstrated that multidetector CT can reveal intricate details of lung complexity and gas distribution, thereby providing deeper insights into reptilian respiratory adaptations (Ricardo 2023).

The rigid and unyielding nature of the chelonian shell imposes unique physiological constraints on the respiratory system, as the lungs must function within a fixed coelomic volume alongside other visceral organs. Understanding the precise anatomical shifts during head and limb retraction is not merely a matter of morphological interest but is of critical clinical and biological importance. From a biological perspective, these adaptations reflect a sophisticated evolutionary trade‐off: the ability to seek total protection within the shell must not compromise gas exchange or lead to airway occlusion. Quantifying how the lung parenchyma and bronchial architecture respond to these internal pressure changes provides deep insights into the respiratory resilience of *T. graeca*. From a clinical standpoint, as tortoises are highly susceptible to lower respiratory tract infections (such as pneumonia), accurate diagnostic imaging is paramount. Since many clinical examinations and CT scans may be performed while the animal is partially or fully retracted due to stress or illness, establishing a baseline of ‘normal’ positional changes is essential. Without these reference metrics, clinicians might misinterpret postural lung compression as pathological consolidation or space‐occupying lesions. Therefore, this study aims to provide the necessary morphometric standards to enhance the accuracy of non‐invasive diagnostics and surgical planning in chelonian medicine.

In light of the existing knowledge gaps, we formulated the following hypotheses for the present study: (1) That limb and neck retraction leads to a localized reduction in lung volume, primarily affecting the cranial region; (2) that lung parenchymal thickness remains invariant despite postural changes; and (3) that the bronchial system adapts to neck retraction through spatial reorientation of its flexures rather than caudal displacement of the tracheal bifurcation.

Despite significant progress, a comprehensive, species‐specific computed tomographic (CT) analysis of the lung morphology in *T. graeca* remains absent from the literature. While CT has proven effective in evaluating the respiratory systems of other reptilian species, the distinctive anatomical features and physiological adaptations of the spur‐thighed tortoise warrant targeted investigation. A detailed understanding of its respiratory architecture is crucial, both for elucidating evolutionary adaptations and for its direct clinical applications. Therefore, this study seeks to characterize the precise anatomical position and three‐dimensional organization of the lower respiratory tract including the trachea, bronchi and lungs in *T. graeca* and to delineate their spatial relationships with adjacent organs in vivo. The results are expected to provide a valuable anatomical reference for clinical diagnostics and to contribute to the broader understanding of reptilian pulmonary morphology.

## 2 Materials and Methods

Ten adult male *T. graeca* tortoises with a mean body weight of 1.65 ± 0.22 kg were used in this study. These turtles were collected from the plains around Ashtian County, Markazi Province, with the coordination of the Iranian Environmental Protection Organization. All 10 samples selected for this study were clinically examined and their health was confirmed. Given that this study focuses on the respiratory system, respiratory health has been of great concern in this subject.

This study was a DVM thesis and all experimental procedures were approved by the Faculty of Veterinary Medicine, University of Tehran (registration number: 30704/6/10). It should be noted that all of the turtles studied survived after the imaging procedures, regained consciousness without any problems, and were returned to their original location.

CT examinations were performed using a Somatom Spirit 2 scanner (Siemens, Germany) with the following technical parameters: A rotation time of 1 s, slice thickness of 1 mm, reconstruction interval of 0.5–1 mm, pitch of 1, x‐ray tube potential of 120 kV and tube current of 130 mA. Appropriate window width (WW) and window level (WL) settings were selected for each anatomical region, with specific values provided in the Results section. Lung window settings were used for image evaluation, and various presets. Also detailed in the corresponding figure captions were applied for 3D reconstructions.

Morphometric measurements were performed using the Syngo MMWP VE40A software installed on the Somatom Spirit 2 CT unit. Several parameters were measured in two conditions: When the head, neck and limbs were fully retracted into the shell, and when they were extended outside the shell. The measured parameters included the following:

1. When the head, neck and limbs were extended outside (protracted) the shell, the following measurements were obtained: The length of the right and left lungs; the width of the right and left lungs in the cranial, middle and caudal regions; the height of the right and left lungs in the cranial, middle and caudal regions; the volume of the right and left lungs; and the parenchymal thickness of the right and left lungs in the cranial, middle and caudal regions.
2. When the head, neck and limbs were fully retracted (flexed) into the shell, the following measurements were obtained: The length of the right and left lungs; the width of the right and left lungs in the cranial, middle and caudal regions; the height of the right and left lungs in the cranial, middle and caudal regions; the volume of the right and left lungs; and the parenchymal thickness of the right and left lungs in the cranial, middle and caudal regions.

The selection of parameters was based on similar studies conducted on other turtle species, such as studies conducted on the European pond turtle (Zehtabvar et al. 2014) and the Caspian pond turtle (Davari et al. 2020).

The following is an explanation of the measured parameters:

- Lung length: Maximum lung length measured in both retracted and extended positions; in Coronal CT reconstruction; bilateral measurement.
- Lung width: Width measured at cranial, middle and caudal regions in both positions; in axial CT images; bilateral measurement.
- Lung height: Height measured at cranial, middle and caudal regions in both positions; in axial CT images; bilateral measurement.
- Lung volume: Right and left lung volumes measured in both positions; in multi‐planar (axial, sagittal, coronal); bilateral measurement, volumetric analysis.
- Coelomic cavity volume: Total coelomic cavity volume; in multi‐planar (axial, sagittal, coronal); bilateral measurement; volumetric analysis.
- Lung parenchymal thickness: Parenchymal thickness at cranial, middle and caudal regions adjacent to dorsal lung wall; in axial CT; bilateral measurement.

Statistical analysis was performed using SPSS software (version 26). Descriptive data were expressed as mean ± standard error of the mean (Mean ± SEM). For data analysis, one‐way ANOVA followed by Tukey post hoc test and an independent *t*‐test were used. A *p*‐value of ≤ 0.05 was considered statistically significant.

## 3 Results

In this study, the topography and positioning of the lower respiratory tract—including the lungs, bronchi and trachea—were evaluated in *T. graeca* using CT imaging and morphometric analysis, and the anatomical relationships between these structures were identified. The following section presents a detailed description of these findings. In Clip S1, the arrangement of the lungs and respiratory canals can be observed in different conditions.

### 3.1 CT Image Evaluation

#### 3.1.1 Position of the Respiratory Structures When the Limbs, Head and Neck Were Extended Outside the Shell

**FIGURE 1:**
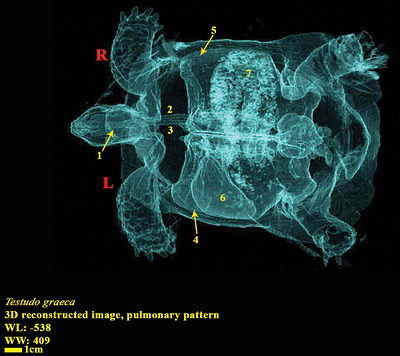
Dorsal view of the three‐dimensional reconstructed CT scan using the Pulmonary pattern in an adult *Testudo graeca*. The image was obtained while the head, neck and limbs were not retracted into the shell. (1) Trachea, (2) right bronchus, (3) left bronchus, (4) left lung, (5) right lung, (6) gas in the stomach, (7) contents of the intestines.

**FIGURE 2:**
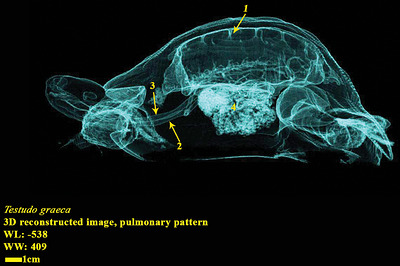
Medial view of the right sagittal slice from the three‐dimensional reconstructed CT scan using the Pulmonary pattern in an adult *Testudo graeca*. The image was obtained while the head, neck and limbs were not retracted into the shell. (1) Right lunge, (2) left bronchus, (3) right bronchus, (4) contents of the intestines.

**FIGURE 3:**
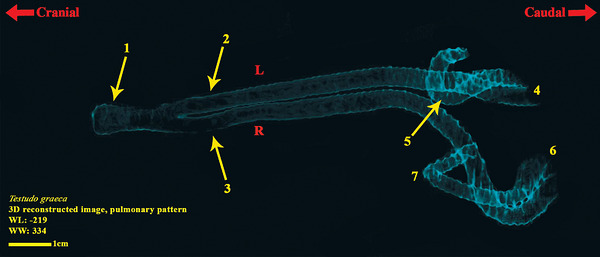
Ventral view of the three‐dimensional reconstructed CT scan using the pulmonary pattern in an adult *Testudo graeca*. In this image, surrounding structures have been removed to better visualize the different parts of the airway. The scan was obtained while the head, neck and limbs were not retracted into the shell. (1) Trachea, (2) left bronchus, (3) right bronchus, (4) proximal flexure (left bronchus), (5) distal flexure (left bronchus), (6) proximal flexure (right bronchus), (7) distal flexure (right bronchus).

In *T. graeca*, the tracheal bifurcation was located within the cervical region and positioned approximately on the ventral aspect of the pharyngeal area, and the trachea itself was relatively short (Figures 1 and 2). The bifurcation was situated near the second and third cervical vertebrae. Both the right and left bronchi coursed toward the right side of the neck as they entered the coelomic cavity (Figures 1 and 3). Just caudal to their entry point into the lungs, both bronchi formed an almost transverse curve and then redirected cranially (Figure 3). After a short distance, each bronchus created a more vertical curve with a caudodorsal orientation before entering the right and left lungs (Figure 3). The left bronchus was longer than the right bronchus (Figure 3).

**FIGURE 4:**
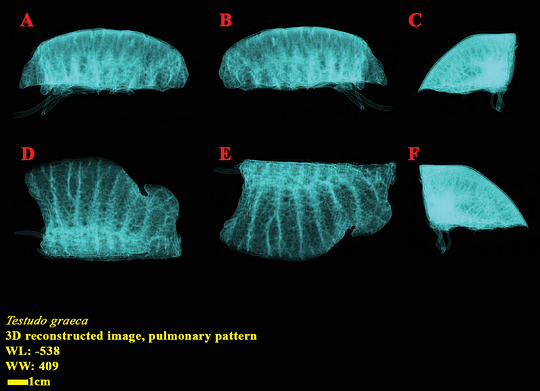
Various views of the three‐dimensional reconstructed CT scan using the pulmonary pattern showing the right lung of an adult *Testudo graeca*. The scan was obtained while the head, neck and limbs were not retracted into the shell. (A) Medial view, (B) lateral view, (C) cranial view, (D) dorsal view, (E) ventral view, (F) caudal view.

Each bronchus formed two distinct curvatures: A proximal bend and a distal bend. The proximal bend was located more caudally, while the distal bend was positioned more cranially (Figures 3 and 4). The distal bend was closer to the point of entry into the lung (Figure 3). The caudal bend was oriented almost transversely and horizontally, whereas the cranial bend was nearly vertical (Figure 3). Each bronchus consisted of three segments: The first segment extended from the tracheal bifurcation to the proximal bend; the second segment extended from the proximal bend to the distal bend; and the third segment extended from the distal bend to the bronchial entry into the lung (Figure 3). Among these three segments, the first was the longest, followed by the second, while the third segment was the shortest (Figure 3).

The proximal bend of the right bronchus was located between the heads of the fourth and fifth ribs, closer to the head of the fourth rib. The proximal bend of the left bronchus was positioned between the heads of the third and fourth ribs, closer to the head of the fourth rib, and it was slightly more cranial than the right proximal bend. The distal bend of the right bronchus was located between the heads of the second and third ribs, closer to the head of the second rib. The distal bend of the left bronchus was also positioned between the heads of the second and third ribs, closer to the head of the second rib. The term ‘rib head’ refers to the articulation of the ribs with the vertebral column as observed in transverse CT images. The entry point of the right bronchus into the lung was located between the heads of the second and third ribs, nearer to the head of the third rib. The entry point of the left bronchus into the lung was also positioned between the heads of the second and third ribs, closer to the head of the third rib and was slightly more caudal than that of the right bronchus.

#### 3.1.2 External Morphology of the Lungs in *T. graeca* When the Limbs, Head and Neck Were Extended Outside the Shell

In *T. graeca*, the lungs had two main surfaces: A dorsal surface positioned against the carapace and a ventral surface adjacent to the visceral organs. When the limbs, head and neck were extended outside the shell, each lung displayed four primary borders: Cranial, caudal, lateral and medial. The overall shape of the lungs was approximately rectangular. The lateral and medial borders were longer than the cranial and caudal borders (Figure 4). The caudal portion of the lateral border showed a distinct notch, and the lung width in the caudal region was generally less than in the cranial region. Both the cranial and caudal borders had a slightly concave appearance (Figure 4).

#### 3.1.3 External Morphology of the Lungs in *T. graeca* When the Limbs, Head and Neck Were Retracted Into the Shell

In this position, compared with the state in which the limbs, head and neck were extended outside the shell, the cranial and caudal borders were less concave, the caudal notch on the lateral border became shallower, and the medial border showed a slight indentation due to the head and neck being positioned between the two lungs. In the cranial part of the lateral border, the curvature was also reduced (Figure 8).

### 3.2 Position of the Respiratory Structures When the Limbs, Head and Neck Were Retracted Into the Shell

**FIGURE 5:**
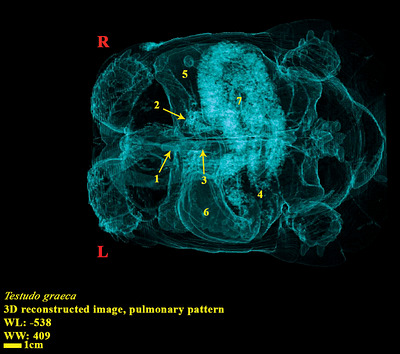
Dorsal view of the three‐dimensional reconstructed CT scan using the pulmonary pattern in an adult *Testudo graeca*. The scan was obtained while the head, neck and limbs were retracted inside the shell. (1) Trachea, (2) right bronchus, (3) left bronchus, (4) left lung, (5) right lung, (6) gas in the stomach, (7) contents of the intestines.

**FIGURE 6:**
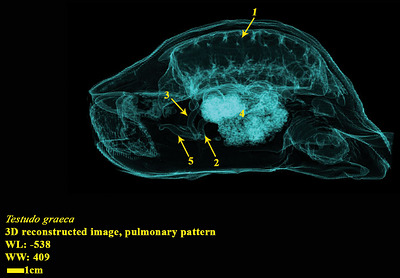
Medial view of the right sagittal slice from the three‐dimensional reconstructed CT scan using the pulmonary pattern in an adult *Testudo graeca*. The scan was obtained while the head, neck and limbs were retracted inside the shell. (1) Right lunge, (2) left bronchus, (3) right bronchus, (4) contents of the intestines, (5) trachea.

**FIGURE 7:**
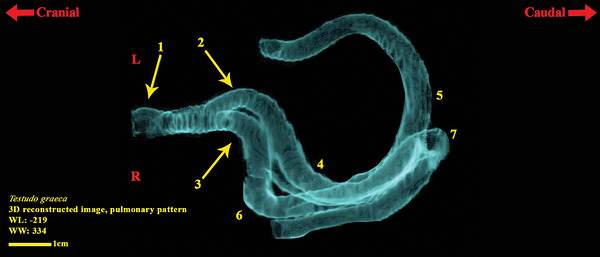
Ventral view of the three‐dimensional reconstructed CT scan using the pulmonary pattern in an adult *Testudo graeca*. Extra structures have been removed to clearly display the different components of the airways. The scan was obtained while the head, neck and limbs were retracted inside the shell. (1) Trachea, (2) left bronchus, (3) right bronchus, (4) proximal flexure (left bronchus), (5) distal flexure (left bronchus), (6) proximal flexure (right bronchus), (7) distal flexure (right bronchus).

The tracheal bifurcation was located in the cervical region and positioned approximately on the ventral aspect of the pharyngeal area. In this posture, with the limbs, head and neck retracted into the shell, both bronchi initially lay on the right side of the neck after the tracheal bifurcation and extended caudally (Figures 5 and 6). The bifurcation was situated near the first and second cervical vertebrae. Shortly after the bifurcation, the right bronchus formed a transverse curve toward the lateral and dorsal directions, then continued caudodorsally with a slight medial inclination (Figure 7). It then created another curve ventrolaterally before turning cranially and finally entering the right lung (Figure 5).

**FIGURE 8:**
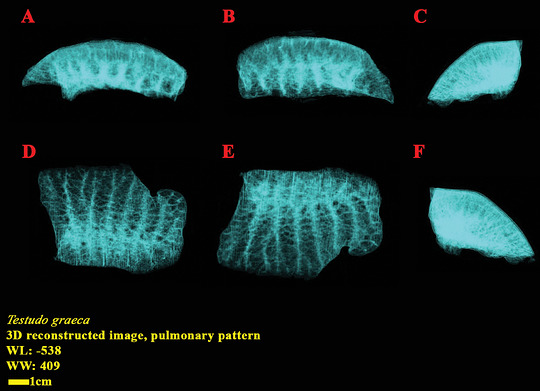
Various views of the three‐dimensional reconstructed CT scan using the pulmonary pattern showing the right lung of an adult *Testudo graeca*. The scan was obtained while the head, neck and limbs were retracted inside the shell. (A) Medial view, (B) lateral view, (C) cranial view, (D) dorsal view, (E) ventral view, (F) caudal view.

After the tracheal bifurcation, the left bronchus extended caudolaterally and became more inclined toward the right side of the neck. It then formed a transverse curve toward the right lateral aspect of the neck and continued caudally. In the caudal part of the neck, it created a transverse curve toward the left side and subsequently travelled cranially on the left side of the coelomic cavity, where it entered the left lung (Figure 7).

The proximal bend of the right bronchus was located between the heads of the second and third ribs on the right side, closer to the head of the third rib. The proximal bend of the left bronchus was also positioned between the heads of the second and third ribs on the right side, closer to the head of the third rib and was slightly more caudal than the right proximal bend.

The distal bend of the right bronchus was located between the heads of the fourth and fifth ribs on the right side, closer to the head of the fifth rib. The distal bend of the left bronchus was positioned between the heads of the fourth and fifth ribs on the left side, closer to the head of the fifth rib and was slightly more cranial than the right distal bend.

The entry point of the right bronchus into the lung was located between the heads of the second and third ribs, nearer to the head of the third rib. The entry point of the left bronchus into the lung was also positioned between the heads of the second and third ribs, closer to the head of the third rib and was slightly more caudal than the right bronchial entry.

### 3.3 Internal Structure of the Lungs

**FIGURE 9:**
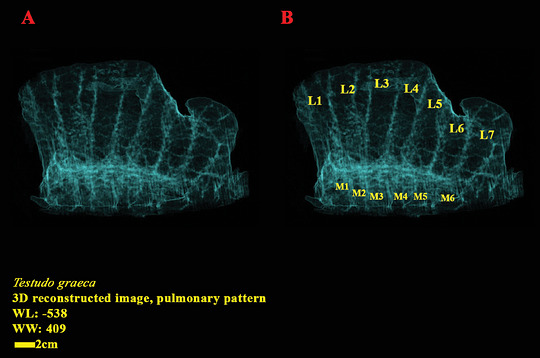
Dorsal view of the three‐dimensional reconstructed CT scan using the pulmonary pattern showing the right lung of an adult *Testudo graeca*. The scan was obtained while the head, neck and limbs were not retracted into the shell. (A) It illustrates the lung without internal chamber labeling. (B) It shows the labeled internal chambers: Lateral chambers (L1–L7) and medial chambers (M1–M6).

In both the right and left lungs, seven lateral chambers and six medial chambers were identified (Figure 9). The lateral chambers were positioned dorsolaterally, while the medial chambers were located ventromedially. In addition, the most cranial lateral chamber extended slightly toward the medial side, and because no separating wall was present between the medial and lateral portions, this region was considered a shared, continuous structure. A similar arrangement was also observed in the most caudal lateral chamber.

**FIGURE 10:**
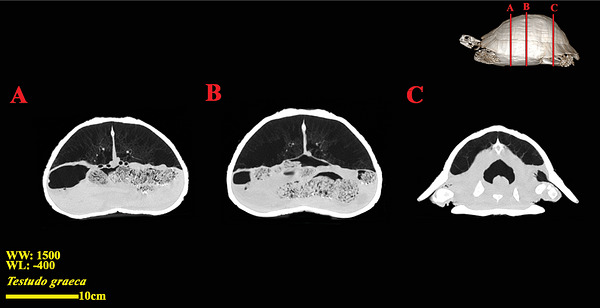
Transverse views of the CT scan of an adult *Testudo graeca* using the pulmonary window, showing parenchymal thickness in different regions of the lung. Images A, B and C represent sequential cranial‐to‐caudal sections of the lungs. The scan was obtained while the head, neck and limbs were not retracted into the shell.

**FIGURE 11:**
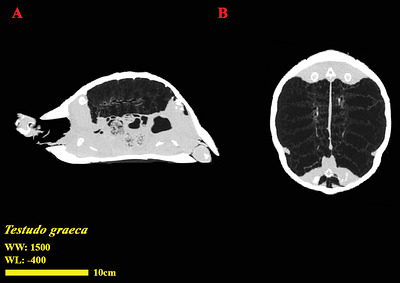
Left sagittal (A) and dorsal (B) views of the CT scan of an adult *Testudo graeca* using the pulmonary window. In image A, the principal bronchus and several secondary bronchi supplying the lateral and medial chambers are visible. In image B, the lateral and medial lung chambers are clearly identified. The scan was obtained while the head, neck and limbs were not retracted into the shell.

After the principal bronchus entered the lung, it extended caudally and gave rise to a secondary bronchus for each chamber, and ultimately the principal bronchus itself terminated in the seventh lateral chamber. The branches supplying the lateral chambers originated dorsally from the principal bronchus (six branches for six lateral chambers), whereas the branches supplying the medial chambers originated ventrally from the principal bronchus (Figures 9, 10, 11).

### 3.4 Morphometric Analysis

The results of the morphometric measurements obtained from the CT examinations of the studied *T. graeca* specimens are presented in Tables 1 and 2.

**TABLE 1:** Descriptive data of the measured indices in the extended (not retracted) and flexed (retracted) positions.

Index	Position	Mean	SEM
Left lung length	Extended	13.53	0.37
	Flexed	13.50	0.37
Right lung length	Extended	13.14	0.37
	Flexed	13.54	0.37
Left lung width (cranial part)	Extended	8.46	0.37
	Flexed	8.11	0.37
Right lung width (cranial part)	Extended	8.52	0.37
	Flexed	8.12	0.37
Left lung width (middle part)	Extended	8.53	0.37
	Flexed	8.37	0.37
Right lung width (middle part)	Extended	8.57	0.37
	Flexed	8.42	0.37
Left lung width (caudal part)	Extended	6.52	0.37
	Flexed	6.48	0.37
Right lung width (caudal part)	Extended	6.56	0.37
	Flexed	6.50	0.37
Left lung height (cranial part)	Extended	5.74	0.37
	Flexed	3.67	0.37
Right lung height (cranial part)	Extended	5.66	0.37
	Flexed	3.46	0.37
Left lung height (middle part)	Extended	6.16	0.37
	Flexed	5.93	0.37
Right lung height (middle part)	Extended	6.03	0.37
	Flexed	6.00	0.37
Left lung height (caudal part)	Extended	4.52	0.37
	Flexed	4.39	0.37
Right lung height (caudal part)	Extended	4.60	0.37
	Flexed	4.30	0.37
Left lung volume	Extended	254.73	2.29
	Flexed	204.88	2.09
Right lung volume	Extended	260.27	1.51
	Flexed	199.69	0.59
Left lung parenchyma thickness (cranial part)	Extended	2.12	0.37
	Flexed	1.97	0.37
Right lung parenchyma thickness (cranial part)	Extended	2.11	0.37
	Flexed	1.97	0.37
Left lung parenchyma thickness (middle part)	Extended	2.17	0.37
	Flexed	2.01	0.37
Right lung parenchyma thickness (middle part)	Extended	2.18	0.37
	Flexed	2.05	0.37
Left lung parenchyma thickness (caudal part)	Extended	1.81	0.37
	Flexed	1.52	0.37
Right lung parenchyma thickness (caudal part)	Extended	1.90	0.37
	Flexed	1.54	0.37

**TABLE 2:** Ratio of total lung volume (right + left) to the coelomic cavity volume in the examined tortoises.

	Lunge Volume/Coelomic cavity volume (%)	
Case	Extended	Flexed
1	34.60	27.18
2	35.50	27.78
3	34.44	27.07
4	34.31	26.98
5	34.21	26.94
Mean	34.61 ± 0.23	27.19 ± 0.15

Using the independent *t*‐test, no significant difference was found in the left lung length between the extended and flexed positions (*p* = 0.956). Similarly, no significant difference was observed in the right lung length between the two positions (*p* = 0.471). Therefore, based on the statistical results, retraction of the limbs, head and neck into the shell does not produce any change in lung length.

Using the independent *t*‐test, no significant difference was observed in the left lung width (cranial part) between the extended and flexed positions (*p* = 0.526). Likewise, no significant difference was found in the right lung width (cranial part) between the two positions (*p* = 0.471).

Using the independent *t*‐test, no significant difference was observed in the left lung width (middle part) between the extended and flexed positions (*p* = 0.770). Similarly, the right lung width (middle part) did not differ significantly between the two positions (*p* = 0.784).

Using the independent *t*‐test, no significant difference was observed in the left lung width (caudal part) between the extended and flexed positions (*p* = 0.941). Likewise, no significant difference was found in the right lung width (caudal part) between the two positions (*p* = 0.912).

Therefore, based on the statistical results, retraction of the limbs, head and neck into the shell does not affect the width of the cranial, middle or caudal regions of the lungs.

Using the independent *t*‐test, a significant difference was observed in the left lung height (cranial part) between the extended and flexed positions (*p* = 0.004). A significant difference was also found in the right lung height (cranial part) between the two positions (*p* = 0.003).

Using the independent *t*‐test, no significant difference was observed in the left lung height (middle part) between the extended and flexed positions (*p* = 0.675). Similarly, no significant difference was found in the right lung height (middle part) between the two positions (*p* = 0.956).

Using the independent *t*‐test, no significant difference was observed in the left lung height (caudal part) between the extended and flexed positions (*p* = 0.812). Likewise, the right lung height (caudal part) did not differ significantly between the two positions (*p* = 0.586).

Therefore, based on the statistical results, retraction of the limbs, head and neck into the shell reduces the height of the cranial region of the lungs, but does not affect the height of the middle or caudal regions.

Using the independent *t*‐test, a significant difference was observed in the left lung volume between the extended and flexed positions (*p* < 0.001). A significant difference was also found in the right lung volume between the two positions (*p* < 0.001).

Therefore, based on the statistical results, retraction of the limbs, head and neck into the shell leads to a reduction in lung volume.

Using the independent *t*‐test, no significant difference was observed in the left lung parenchyma thickness (cranial part) between the extended and flexed positions (*p* = 0.784). Likewise, no significant difference was found in the right lung parenchyma thickness (cranial part) between the two positions (*p* = 0.797).

Using the independent *t*‐test, no significant difference was observed in the left lung parenchyma thickness (middle part) between the extended and flexed positions (*p* = 0.770). Similarly, the right lung parenchyma thickness (middle part) did not differ significantly between the two positions (*p* = 0.812).

Using the independent *t*‐test, no significant difference was observed in the left lung parenchyma thickness (caudal part) between the extended and flexed positions (*p* = 0.598). Likewise, no significant difference was found in the right lung parenchyma thickness (caudal part) between the two positions (*p* = 0.515).

Therefore, based on the statistical results, retraction of the limbs, head and neck into the shell does not produce any change in parenchymal thickness in the cranial, middle or caudal regions of the lungs.

Using one‐way ANOVA, no significant differences were observed in left parenchyma thickness (extended position) among the cranial, middle and caudal regions (*p* = 0.766). Similarly, in the flexed position, no significant differences were found among these regions for left parenchyma thickness (*p* = 0.601).

Using one‐way ANOVA, no significant differences were observed in right parenchyma thickness (extended position) among the cranial, middle and caudal regions (*p* = 0.860). Likewise, in the flexed position, no significant differences were detected among these regions for right parenchyma thickness (*p* = 0.595).

Based on these findings, parenchymal thickness did not differ among cranial, middle and caudal regions of either the right or left lung in both extended and flexed positions.

Using an independent *t*‐test, a significant difference was observed in the mean ratio of total lung volume to coelomic cavity volume between the extended and flexed positions (*p* < 0.001).

According to the statistical results, flexion of the limbs, head and neck within the shell reduces the percentage of the coelomic cavity occupied by the lungs. In the extended position, 34.61% of the coelomic cavity was occupied by the lungs, whereas in the flexed position this value decreased to 27.19% (Table 2).

## 4 Discussion

### 4.1 Tracheal and Bronchial Positioning in Turtles

The anatomical positioning of the trachea and primary bronchi varies considerably among chelonian species, often correlating with neck mobility and ecological niche.

In sea turtles, which are incapable of neck retraction, the tracheal anatomy reflects their aquatic lifestyle. For example, in the loggerhead sea turtle (*C. caretta*), the trachea runs ventral and slightly to the right of the oesophagus (Valente et al. 2007), and its bifurcation is situated more caudally compared to that of terrestrial tortoises—a feature that does not impose a respiratory limitation in these non‐retractile species (Murray 2006).

In contrast, turtles capable of cervical retraction exhibit anatomical adaptations to ensure continuous airflow when the neck is withdrawn. A key adaptation among cryptodiran turtles is a relatively short trachea, which facilitates easier neck retraction (Wyneken 2001). Interestingly, some species combine this with a caudally positioned bifurcation. The European pond turtle (*Emys orbicularis*), for instance, possesses a relatively long trachea with a caudal bifurcation located on the left side of the coelomic cavity. This specific configuration is considered an adaptation for maintaining respiration when the neck is fully retracted into the shell (Zehtabvar et al. 2014). Similarly, in the common snapping turtle (*Chelydra serpentina*), the trachea courses along the left side of the neck before bifurcating within the coelom into the primary bronchi, with the left bronchus being notably shorter (Schachner et al. 2017).

In our study on spur‐thighed tortoises (*T. graeca*), the tracheal bifurcation was located in the cervical region at the ventral aspect of the pharyngeal area. The trachea was short in length, and the bifurcation was positioned near the second and third cervical vertebrae. In this species, the trachea does not deviate markedly to one side; instead, after bifurcation, both primary bronchi shift together toward the right side of the neck. This configuration appears to prevent airway obstruction when the head and neck are withdrawn into the shell.

In *T. graeca*, the entry point of the right and left bronchi into the lungs remained constant in both extended and flexed postures of the limbs and neck, and it was the course and orientation of the bronchi themselves that changed between postures. An important observation in this species is the presence of both proximal and distal bronchial bends in all positions. In contrast, in other species, these bends have not been described when the limbs and neck are not withdrawn into the shell. A study on the European pond turtle (*E. orbicularis*) reported that when the neck is retracted to the point that the tracheal bifurcation lies caudal to the bronchial entry into the lungs, a bend develops along the bronchial path (Zehtabvar et al. 2014).

### 4.2 Lung Structure in Turtles

In the *T. graeca*, as in other turtle species, the lungs are paired structures and their internal surface has a reticulated appearance. In addition, the lungs are not situated within a pleural cavity; instead, they are attached dorsally to the carapace (Schwarz and Saunders 2011).

It has been reported that when the neck and limbs are withdrawn, lung volume may decrease to as much as one‐fifth of its original size. In our study, we also observed that during neck retraction, the cervical region shifts between the lungs and contributes to a reduction in their volume (Mitchell 2009). In the Caspian pond turtle (*Mauremys capsica*), it has been noted that when the head, neck and limbs are extended, the lungs occupy 42.12% of the coelomic cavity, whereas this value decreases to 22.58% in the flexed posture. In the present study, the lungs occupied 34.61% of the coelomic cavity in the extended position and 27.19% in the flexed position (Davari et al. 2020). It should be considered that the *M. capsica* is a semi‐aquatic species, whereas *T. graeca* is fully terrestrial. The difference in coelomic occupancy between these species may be related to the fact that semi‐aquatic turtles, such as the Caspian pond turtle, rely on the air within their lungs to maintain flotation while swimming. Therefore, under normal conditions, a larger proportion of their coelomic cavity is filled with air compared with terrestrial species such as *T. graeca*.

In studies conducted on the European pond turtle (*E. orbicularis*), it has been reported that when the neck and limbs are retracted, two major changes occur: Compression and ventrolateral displacement of the apical regions of the lungs, and separation of the medial lung surfaces from one another (Zehtabvar et al. 2014). In the spur‐thighed tortoise (*T. graeca*), an apical lung structure was not identified; however, when the head and neck were withdrawn into the shell, the cranial lung margins displayed reduced indentation, the medial borders became slightly concave, and the caudal notch along the lateral margin became shallower. Based on the statistical measurements of lung length, width and height in this species, it can be inferred that the reduction in lung volume during retraction of the limbs, head and neck is likely due to a decrease in the height of the cranial portion of the lungs.

In chelonians, lung morphology exhibits both shared structural patterns and notable interspecific variations. For instance, in the semi‐aquatic common snapping turtle (*C. serpentina*), the symmetrical, wedge‐shaped lungs occupy approximately two‐thirds of the dorsal coelomic cavity (Schachner et al. 2017). A similar wedge shape has been reported for another semi‐aquatic species, the European pond turtle (*E. orbicularis*) (Zehtabvar et al. 2014). In contrast, the lungs of the fully terrestrial spur‐thighed tortoise (*T. graeca*) present a generally rectangular profile, tapering from a broader cranial to a narrower caudal portion.

The bronchial architecture reveals a fundamental branching pattern conserved across these species. In *C. serpentina*, secondary bronchi arise laterally and medially from the principal (intrapulmonary) bronchus (Schachner et al. 2017), a pattern also confirmed in *T. graeca* in the present study. However, quantitative differences exist. While *C. serpentina* typically exhibits three lateral and three medial secondary bronchi, and the alligator snapping turtle (*Macrochelys temminckii*) four of each (Lambertz et al. 2010), our investigation of *T. graeca* identified six secondary bronchi in each orientation, with the principal bronchus extending into a seventh lateral chamber.

Internal chamber organization further distinguishes these species. In *C. serpentina*, each lung contains three smaller medial and three larger lateral chambers, all interconnected via bronchi, along with a distinct terminal caudal chamber and a specialized medially‐oriented tertiary bronchus. Conversely, in *T. graeca*, we observed seven lateral and six medial chambers, with the lateral chambers being larger a size relationship consistent with *C. serpentina* but no terminal chamber was identified (Schachner et al. 2017). These morphological divergences may reflect ecological adaptations, as *T. graeca* is fully terrestrial, whereas *C. serpentina* and *E. orbicularis* are semi‐aquatic (Ernst 2014; Zehtabvar et al. 2014).

The evolution of aspiration breathing in tetrapods involved a fundamental shift of ventilatory responsibility from the hyobranchial system to the axial musculoskeletal system, with the transverse abdominal (TA) muscle emerging as a primitive character for active exhalation (Brainerd and Owerkowicz 2006). Our findings regarding the structural stability of the turtle's airways during defensive contraction highlight an evolutionary innovation that circumvents the ‘axial constraint’—a mechanical conflict between locomotion and costal ventilation commonly observed in other reptiles (Boggs 2002). Unlike many squamates that experience compromised tidal volumes during body wall activity, turtles have evolved specialized muscular and structural arrangements that decouple ventilatory cycles from axial body movements (Brainerd and Owerkowicz 2006; Boggs 2002). This physiological independence, evidenced by the preserved patency of the airways in our CT data despite coelomic compression, suggests that turtles maintain the structural integrity of their respiratory system to ensure continuous gas exchange during defensive postures.

Furthermore, the structural simplicity of the turtle lung does not preclude advanced aerodynamic functions. Recent discoveries in iguanas and monitor lizards have demonstrated that unidirectional airflow can occur in non‐parabronchial lungs through aerodynamic valving and high‐velocity jets (Cieri et al. 2014; Schachner et al. 2014). Our results indicate that the maintenance of bronchial geometry during defensive contraction provides the necessary conduit for such aerodynamic patterns to persist. Concurrently, local control of pulmonary blood flow and the presence of hypoxic pulmonary vasoconstriction (HPV) in multicameral lungs optimize ventilation‐perfusion matching even in compressed regions (Skovgaard and Wang 2006). Interestingly, the pressure oscillations induced by defensive body contractions may not hinder respiration; instead, they might facilitate convective mixing and enhanced oxygen diffusion, similar to high‐frequency oscillation mechanisms observed in other tetrapods (Boggs 2002; Skovgaard and Wang 2006).

The findings of the present study demonstrate that CT provides a detailed and reliable evaluation of the respiratory system in *T. graeca*, allowing assessment of lung volume changes across different body positions without alterations in the overall bronchial pattern or lung morphology. These results are consistent with previous studies in other chelonian species, which have shown that CT, as a non‐invasive imaging modality, enables precise visualization of intrapulmonary bronchi, associated vasculature and the multicameral organization of the pulmonary parenchyma (Andrés Polanco et al. 2020). In contrast, although radiography is commonly used for the evaluation of respiratory diseases in turtles, its diagnostic value is limited due to the superimposition of the carapace and plastron, which obscures pulmonary structures and reduces the accuracy of parenchymal assessment (Christopher and Hernandez‐Divers 2003). These limitations highlight the diagnostic superiority of CT for evaluating the respiratory system in chelonians, particularly in species such as spurred tortoises, where internal organs are enclosed within a rigid shell.

Furthermore, the present study revealed that changes in body position—specifically retraction of the head, neck and limbs—result in a significant reduction in lung volume, especially in the cranial regions, while the overall anatomical structure of the lungs remains unchanged. This observation is in agreement with previous studies in other turtle species, which have demonstrated that lung volume and its proportion within the coelomic cavity are strongly influenced by body posture (Davari et al. 2020; Mitchell 2009). In addition, imaging‐based studies have shown that the lungs of turtles exhibit a reticular, multicameral architecture without clear lobation, features that are well visualized using CT (Duncker 2004). In this context, CT not only facilitates accurate anatomical assessment but also serves as a critical diagnostic tool for detecting lesions that may not be visible on conventional radiographs (Mackey et al. 2008). Therefore, the results of the present study further support the value of CT as an advanced imaging modality for the evaluation of the respiratory system in chelonians and emphasize the importance of considering body position when interpreting imaging findings and assessing respiratory conditions.

The findings of the present study regarding the use of CT for evaluating the respiratory system in *T. graeca* are consistent with the growing application of advanced imaging techniques in reptile medicine. In recent years, CT has been recognized as the modality of choice for pulmonary assessment in chelonians, as it enables detailed evaluation of pulmonary parenchyma, as well as quantitative measurements such as lung volume and tissue attenuation values (Hounsfield units) (Da Silva et al. 2020). In contrast to conventional radiography, which is limited by the superimposition of the carapace and plastron, CT provides cross‐sectional imaging and multiplanar reconstructions that allow precise visualization of internal structures. Previous studies have demonstrated that CT is not only useful for describing normal anatomy but also for establishing baseline reference values for lung density and volume, which are essential for the diagnosis of respiratory diseases (Da Silva et al. 2020).

Moreover, the role of CT in the detection of pathological pulmonary conditions has been well documented in reptiles. For instance, in a reported case of pulmonary fibrosis in a leopard tortoise, CT imaging revealed interstitial thickening, ground‐glass opacity and honeycombing patterns that were not clearly identifiable on radiographs (Lim et al. 2013). These findings highlight the superior sensitivity of CT in detecting subtle parenchymal changes. In addition, comprehensive reviews on reptile imaging have emphasized that the presence of the shell significantly limits radiographic interpretation in chelonians, making CT the preferred modality for evaluating the coelomic cavity and respiratory system (Gumpenberger 2021). It has also been noted that mechanical compression of the lungs in turtles may mimic pathological conditions such as pneumonia on imaging, underscoring the importance of understanding normal respiratory anatomy and physiology when interpreting CT findings (Gumpenberger 2021). Collectively, these observations support the use of CT in the present study as both an anatomically accurate and clinically relevant tool, aligned with current standards in diagnostic imaging of reptiles.

Furthermore, considering the connection between the principal bronchus and the seventh lateral chamber in the spur‐thighed tortoise, and the connection of this bronchus to the terminal chamber in the common snapping turtle (*C. serpentina*), it may be inferred that these two structures share certain evolutionary relationships. Although the parenchyma in this region is well developed in *T. graeca*, the parenchyma of the terminal chamber in *C. serpentina* is much less substantial compared with the more cranial regions (Schachner et al. 2017). In the present study on *T. graeca*, six secondary bronchi were observed to arise dorsally from the principal bronchus to supply the six lateral chambers, while the seventh lateral chamber communicated directly with the principal bronchus. In addition, six secondary bronchi supplying the medial chambers originated ventrally from the principal bronchus. It should also be noted that in *T. graeca*, the parenchymal thickness in the cranial, middle and caudal regions of the lung remained consistent and did not exhibit the caudal thinning described in some other species.

Comparative analysis between Testudo graeca and Emys orbicularis indicates that although both species exhibit a similar functional response of the respiratory system to postural changes, the magnitude of morphometric alterations differs considerably. In Emys orbicularis, total lung volume decreased significantly by approximately 20‐22% during flexed positioning, whereas in Testudo graeca the reduction was less pronounced, ranging between 15‐18%. Similarly, cranial regional narrowing and displacement were more evident in Emys orbicularis, while Testudo graeca showed a relatively more stable cranial lung configuration. Parenchymal thickness variability also differed between the two species, with Emys orbicularis demonstrating up to 25‐30% regional differences, particularly between cranial and caudal areas, compared to the more limited variation of approximately 10‐15% observed in Testudo graeca. These findings suggest that although both species share a conserved biomechanical strategy for accommodating body posture changes, Emys orbicularis exhibits greater morphofunctional plasticity in response to coelomic compression, likely reflecting species‐specific ecological and locomotor adaptations (Zehtabvar et al., 2026).

Comparative analysis of Testudo graeca and Emys orbicularis shows that both species possess a multicameral lung architecture; however, significant differences exist in the degree of internal subdivision and chamber complexity. In the European pond turtle, the lungs contain multiple lateral and medial chambers, as well as a relatively large terminal (abdominal) chamber that plays an important role in air storage. In this species, the separation of compartments is more distinct, and the boundaries between lateral and medial chambers are clearly defined. In contrast, in the spur‐thighed tortoise (Testudo graeca), although the general pattern of compartmentalization is similar, the chambers are more uniform, with less distinct separation and a relatively smaller terminal chamber. Furthermore, Emys orbicularis exhibits a greater number and clearer differentiation of medial chambers compared to lateral ones, resulting in a more complex spatial organization, whereas Testudo graeca demonstrates a more compact lung structure with less variability in the size and shape of internal spaces. These observed differences in the internal lung structure and the degree of chamber subdivision between the two species may be related to differences in their lifestyle and environmental conditions. The European pond turtle (Emys orbicularis) is a semi‐aquatic species that spends a considerable portion of its life cycle in aquatic environments such as ponds and wetlands, whereas the spur‐thighed tortoise (Testudo graeca) is a fully terrestrial species. This ecological distinction is likely to have influenced the evolution of pulmonary ventilation patterns, air storage requirements, and the complexity and compartmentalization of internal lung chambers, which may explain the differences observed in the size, clarity, and organization of intrapulmonary spaces between these two species.

## 5 Conclusion

The findings of the present study lead to the following conclusions based on our initial hypotheses:

1. Confirmed: Lung volume reduction is statistically significant and localized to the cranial region, as evidenced by the significant decrease in cranial lung height.
2. Confirmed: Lung parenchymal thickness is invariant and unaffected by postural changes, ensuring consistent structural integrity for gas exchange.
3. Confirmed: The respiratory system accommodates neck retraction through the geometric flexibility of bronchial flexures rather than the displacement of the tracheal bifurcation.

These results offer valuable insights into the functional morphology of the lower respiratory tract in *T. graeca* and emphasize the high diagnostic value of CT imaging in chelonian medicine.

These findings clearly support our initial hypotheses, demonstrating that *T. graeca* accommodates its respiratory structures within the limited coelomic space through localized volume reduction and bronchial reorientation, while maintaining the structural integrity of the lung parenchyma.

Based on all the observations in *T. graeca*, it can be concluded that during the process of neck retraction, the lungs are compressed, and the bifurcation of the trachea does not move caudally. The orientation of the bronchi leading to the lungs changes. Furthermore, the reduction in lung volume when the limbs, head and neck are retracted occurs due to a decrease in the cranial part of the lung's height as the head and neck position between the two lungs. Given the high diagnostic accuracy of CT scans for detecting both normal and abnormal structural features, this method is one of the most effective diagnostic techniques for reptiles. Since the internal structures of turtles are enclosed within their shell, conducting examinations presents certain limitations. Therefore, the use of diagnostic imaging techniques proves to be invaluable in identifying various issues. In this study, the structural characteristics and the positioning of different components of the respiratory system within the coelomic cavity of *T. graeca* were examined, establishing a basis for further investigation and advancing our understanding of reptilian respiratory systems.

## Author Contributions

**Omid Zehtabvar**: conceptualization, investigation, funding acquisition, writing – original draft, methodology, validation, visualization, writing – review and editing, software, formal analysis, project administration, data curation, supervision, resources. **Ali Reza Vajhi**: methodology, writing – original draft. **Amir Rostami**: writing – original draft, methodology. **Hesameddin Akbarein**: conceptualization, investigation, funding acquisition, writing – original draft, methodology, validation, visualization, writing – review and editing, formal analysis, data curation. **Somaye Davudypoor**: software, writing – original draft, data curation. **Zahra Sherafat**: writing – original draft, methodology, software, data curation. **Seyyed Kamyab Momeni**: writing – original draft, methodology, software. **Seyyed Hossein Modarres Tonekabony**: writing – original draft, software. All authors read and approved the final version of the manuscript.

## Funding

The authors have nothing to report.

## Ethics Statement

This study was a DVM thesis and all experimental procedures were approved by the Faculty of Veterinary Medicine (ID: 30704/6/10).

## Conflicts of Interest

The authors declare no conflicts of interest.

## Supporting information




**Supporting File 1: Clip. 1** Three‐dimensional reconstructed CT scan using the pulmonary pattern, adult *Testudo graeca*. In the clip, the following are shown in sequence: the arrangement of the lungs and respiratory canals when the head and limbs are protracted outside the shell; the arrangement of the lungs and respiratory canals in this same condition; the arrangement of the lungs and respiratory canals when the head and limbs are retracted into the shell; and the arrangement of the lungs and respiratory canals in this same condition.

## Data Availability

The data that support the findings of this study are available from the corresponding author upon reasonable request.
